# Understanding cold bias: Variable response of skeletal Sr/Ca to seawater *p*CO_2_ in acclimated massive Porites corals

**DOI:** 10.1038/srep26888

**Published:** 2016-05-31

**Authors:** Catherine Cole, Adrian Finch, Christopher Hintz, Kenneth Hintz, Nicola Allison

**Affiliations:** 1 Department of Earth and Environmental Sciences, University of St. Andrews, St. Andrews KY16 9AL, UK; 2 Department of Marine and Environmental Sciences, Savannah State University, Savannah, GA USA; 3 Department of Electrical and Computer Engineering, George Mason University, Fairfax, VA USA

## Abstract

Coral skeletal Sr/Ca is a palaeothermometer commonly used to produce high resolution seasonal sea surface temperature (SST) records and to investigate the amplitude and frequency of ENSO and interdecadal climate events. The proxy relationship is typically calibrated by matching seasonal SST and skeletal Sr/Ca maxima and minima in modern corals. Applying these calibrations to fossil corals assumes that the temperature sensitivity of skeletal Sr/Ca is conserved, despite substantial changes in seawater carbonate chemistry between the modern and glacial ocean. We present Sr/Ca analyses of 3 genotypes of massive *Porites* spp. corals (the genus most commonly used for palaeoclimate reconstruction), cultured under seawater *p*CO_2_ reflecting modern, future (year 2100) and last glacial maximum (LGM) conditions. Skeletal Sr/Ca is indistinguishable between duplicate colonies of the same genotype cultured under the same conditions, but varies significantly in response to seawater *p*CO_2_ in two genotypes of *Porites lutea*, whilst *Porites murrayensis* is unaffected. Within *P. lutea*, the response is not systematic: skeletal Sr/Ca increases significantly (by 2–4%) at high seawater *p*CO_2_ relative to modern in both genotypes, and also increases significantly (by 4%) at low seawater *p*CO_2_ in one genotype. This magnitude of variation equates to errors in reconstructed SST of up to −5 °C.

Coral skeletal Sr/Ca is influenced by sea surface temperature (SST) and is a widely used palaeothermometer^1^. Strong correlations have been observed between instrumental SST records and skeletal Sr/Ca in modern corals over seasonal to decadal timescales^2^,^3^,^4^, providing a reliable proxy for the reconstruction of multi-centennial SST variations and valuably extending the record of many natural climate oscillations back to the 16^th^ Century^5^,^6^,^7^. Over millennial timescales, the accuracy of SST estimates from Sr/Ca ratios in fossil corals has been questioned as they frequently yield improbably cool or spatially inconsistent values^8^,^9^,^10^. Whilst some of these inconsistencies have been explained by regional oceanographic dynamics^11^, a number of possible influences on skeletal Sr/Ca in coral remain unresolved. Skeletal Sr/Ca is correlated with skeletal growth rate in some^12^,^13^, but not all^14^,^15^ corals and may be influenced by diagenesis^16^,^17^, skeletal ultrastructure^14^,^18^, metabolic cycles^19^, and potential variations in seawater Sr/Ca^20^. Calibration equations describing the temperature dependence of coral Sr/Ca also vary significantly, both between species and within a single species sampled from different reef locations^21^,^22^. However these factors appear insufficient to explain cold bias e.g. in an early Holocene Tahitian fossil coral^10^. Understanding the origin of sensitivity variations within the Sr/Ca-SST relationship is essential if we are to interpret accurately the high resolution climate records encoded in coral skeletons and benefit from their application in resolving the controls on past climates and predicting future change. Coral palaeoproxy relationships are derived by correlating annual SST variations with skeletal Sr/Ca in modern specimens^21^,^22^. In applying these empirical relationships to fossil specimens, it is implicitly assumed that the processes affecting skeletal geochemistry have not changed between the present and past. It is unlikely that this is the case: in particular, atmospheric CO_2_ has varied significantly over glacial-interglacial time scales^23^ affecting both seawater pH and dissolved inorganic carbon (DIC) chemistry^24^, factors which influence skeletal trace element incorporation in some calcitic marine organisms^25^.

The influence of pH and DIC on coral skeletal Sr/Ca is poorly constrained. Culture studies investigating the effect of reduced seawater pH across a range of ocean acidification scenarios yield mixed results: seawater pH and Sr/Ca were negatively correlated in *Acropora digitifera*^26^ and newly settled recruits of *Favia fragum*^27^ but were unrelated in *Montipora capitata*^13^. No systematic variation in skeletal Sr/Ca was found over a wider pH range (both lower and higher than present day) in *Stylophora pistillata*^28^. With the exception of *A. digitifera*^26^, these studies used addition of HCl or NaOH to manipulate seawater pH at constant seawater *p*CO_2_, consequently shifting other carbonate system parameters to unrealistic values^29^. Additionally, the short acclimation times (5–35 days) of three^26^,^27^,^28^ of the four studies are probably insufficient to enable coral physiological responses e.g. changes in zooxanthellae density, to adapt to changes in environment.

We test the direct impact of variations in seawater *p*CO_2_ on skeletal Sr/Ca in massive *Porites* spp. corals, the coral genus most commonly used for palaeoclimate reconstruction. Heads from 3 genetically distinct corals (2× *Porites lutea* and 1× *Porites murrayensis*; Fig. 1) were divided into smaller sub-colonies (each >8 cm diameter) and cultured in a mixture of natural and synthetic seawater. Corals were housed in a purpose-built large-volume aquarium system constructed of low CO_2_ permeability materials. The seawater in each treatment was bubbled with gas mixes set to reach the target seawater *p*CO_2_ compositions. Corals were maintained at ambient *p*CO_2_ conditions for 2 months, adjusted to *p*CO_2_ treatment conditions over another 2 months and then acclimated at the final treatment *p*CO_2_ for 5 months. Final seawater *p*CO_2_ target levels ranged from the last glacial maximum (LGM; ~180 μatm), through present day (~400 μatm) to levels projected by the year 2100 (~750 μatm). Actual seawater *p*CO_2_ levels (198, 416 and 750 μatm) in the reservoirs were calculated from total alkalinity and DIC using the CO2SYS program^30^. Seawater Sr/Ca, temperature, salinity and DIC system parameters were maintained within narrow limits throughout the study (Table 1).

The skeleton deposited in the 5-week period, following the acclimation, was identified by alizarin red staining (Fig. 1), and skeletal Sr/Ca of this region was analysed by secondary ion mass spectrometry (SIMS). Skeletal deposition is concentrated at the skeleton surface in massive *Porites* corals, although subtle thickening of the trabeculae may occur over a period of weeks^31^. The high spatial resolution of SIMS allows the selective analysis of skeleton deposited at the skeletal surface, avoiding the centres of calcification where Sr/Ca is anomalously high, and any material which thickens the trabecula and may be deposited a considerable time later^32^. The accuracy of our SIMS estimates is hindered by uncertainty in the elemental composition of the NaHaxby2 standard, but our analytical precision, which is critical to palaeothermometry, is good (within 0.6%, or 0.018 mmol mol^−1^, (2σ), equivalent to ±0.4 °C).

## Results and Discussion

### Skeletal Sr/Ca varies with seawater *p*CO_2_ in some genotypes

**Figure 2:**
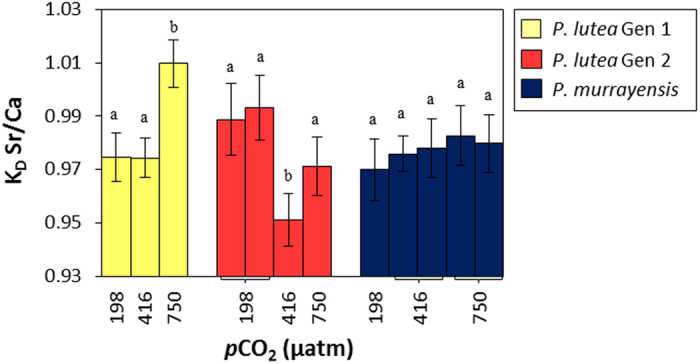
Influence of seawater *p*CO_2_ on coral aragonite:seawater Sr/Ca partition coefficients (K_D_^Sr/Ca^) for 3 genotypes of *Porites* spp. following acclimation to 198, 416 and 750 μatm *p*CO_2_ at 25 °C. Bars represent the mean K_D_^Sr/Ca^ of multiple analyses (n = 12–41; see Table S1) across 2 or more skeletal units within individual colonies. Within each species/genotype, different letters indicate significant differences between treatments (p < 0.05; ANOVA and Tukey post-hoc). Error bars represent the combined 95% confidence limits of seawater and skeletal Sr/Ca measurements.

Coral aragonite Sr/Ca partition coefficients (K_D_^Sr/Ca^ = skeletal Sr/Ca/seawater Sr/Ca) are estimated for each coral (Supplementary Table S1). K_D_^Sr/Ca^ varies significantly in response to perturbations in seawater *p*CO_2_ in both genotypes of *P. lutea* but is unaffected in *P. murrayensis* (Fig. 2). Seawater temperatures do not vary significantly between the treatments (Table 1) and we observe excellent agreement (within 0.3%) in the K_D_^Sr/Ca^ of duplicate sub colonies of the same coral genotype within each treatment (Fig. 2), indicating that minor differences in coral positioning and lighting in each tank do not affect skeletal Sr incorporation. K_D_^Sr/Ca^ is significantly increased at high seawater *p*CO_2_ compared to ambient in both genotypes of *P. lutea* (by 4% in genotype 1, and 2% in genotype 2; *p* < 0.05, ANOVA and Tukey post-hoc). K_D_^Sr/Ca^ is also significantly increased (by 4%; *p* < 0.05) in *P. lutea* genotype 2 at low *p*CO_2_ compared to ambient. In contrast, K_D_^Sr/Ca^ does not vary significantly between the *P. murrayensis* sub-colonies cultured over the full seawater *p*CO_2_ range.

Two hypotheses have been proposed to explain the impact of seawater *p*CO_2_ on coral skeletal Sr/Ca^26^. Coral aragonite precipitates from an extracellular calcifying fluid enclosed in a semi-isolated space between the coral tissue and underlying skeleton, and corals increase the pH of this fluid (upregulate pH) above that of ambient seawater to promote high fluid aragonite saturation states favourable for skeletal precipitation. The coral calcification fluid is derived from seawater which is transported paracellularly (between cells) to the calcification site and from additional Ca transported transcellularly (across cells) via L-type Ca channels^33^,^34^ and the enzyme Ca-ATPase^33^,^35^. Enhancing transcellular Ca transport could decrease the Sr/Ca of the calcification fluid. This hypothesis is appealing as corals maintained at high seawater *p*CO_2_ are able to upregulate calcification fluid pH more to partially offset the effect of lowered seawater pH^36^. Ca-ATPase is a Ca^2+^:H^+^ antiport which increases the pH of the calcification fluid^37^ and may be expected to have a higher activity under high seawater *p*CO_2_. This hypothesis suggests that coral skeletal Sr/Ca is decreased at high seawater *p*CO_2_. However, Sr^2+^ has a similar ionic radius to Ca^2+^ and inhibition of Ca channels and Ca-ATPase in the branching coral, *Pocillopora damicornis*, did not affect skeletal Sr/Ca, suggesting that transmembrane Ca transport does not fractionate Sr/Ca in the calcification fluid^38^.

Alternatively, skeletal Sr/Ca variations may reflect elemental partitioning during aragonite precipitation (Rayleigh fractionation). Rayleigh fractionation occurs when variable volumes of an isolated fluid reservoir are used for solid precipitation, and a trace element ion (in this case Sr) is either discriminated against or preferentially incorporated compared to the major precipitating ion (in this case Ca). The Sr/Ca partition coefficient in aragonite is >1^39^, so Sr is preferentially incorporated into the growing crystals over Ca^40^. As precipitation proceeds, the Sr/Ca of the fluid remaining in the reservoir, and of the carbonate subsequently precipitated from it, decreases. The final Sr/Ca of the aragonite reflects the proportion of the reservoir used in precipitation^40^. The deposition rate of coral aragonite is strongly dependent on the saturation state of the calcifying fluid^41^,^42^. At high calcification fluid saturation states, a higher proportion of the fluid reservoir may be used for calcification resulting in a low skeletal Sr/Ca. Although corals at high seawater *p*CO_2_ increase the pH of the calcification fluid more than their counterparts cultured at ambient *p*CO_2_, they do not ultimately attain such a high fluid pH^36^ and calcification rates in these corals are usually reduced^43^. This hypothesis suggests that coral skeletal Sr/Ca will be increased at high seawater *p*CO_2_. Neither hypothesis immediately explains our observed pattern of skeletal Sr/Ca variations where skeletal Sr/Ca is increased in some corals at both low and high seawater *p*CO_2_. However it is unlikely that the two hypotheses operate independently as the saturation state of the calcification fluid is probably highly responsive to Ca-ATPase activity.

### Growth rate and skeletal Sr/Ca

We explored the relationship between coral aragonite:seawater K_D_^Sr/Ca^ and skeletal growth rate, inferring that Rayleigh fractionation is linked to skeletal precipitation rate. We observe significant differences in both the skeletal extension and calcification rate of some genotypes between treatments (Supplementary Table S2). However, correlation between K_D_^Sr/Ca^ and either skeletal linear extension or calcification rate (Supplementary Fig. S1) are insignificant in all coral genotypes (*p* > 0.3; Pearson’s correlation). Our data suggest that variations in Sr incorporation between corals do not reflect Rayleigh fractionation.

### Alternative controls on skeletal Sr/Ca

**Figure 3:**
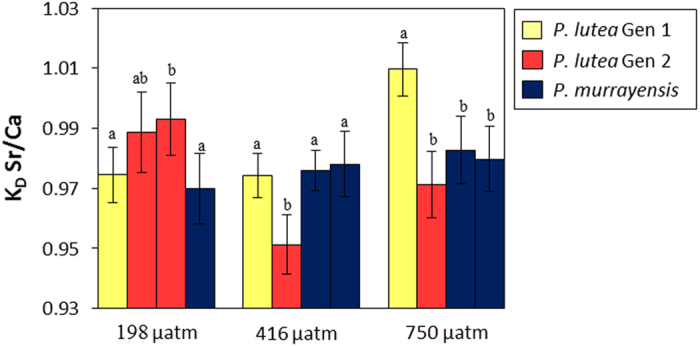
Genotypic variations in mean coral aragonite: seawater K_D_^Sr/Ca^ in individual colonies of *Porites* spp. following >5 months of acclimation to 198, 416 and 750 μatm^3^ *p*CO_2_ at 25 °C. Within each *p*CO_2_ treatment, different letters indicate significant differences between genotypes (p < 0.05; ANOVA and Tukey post-hoc). Error bars represent the combined 95% confidence limits of seawater and skeletal Sr/Ca measurements.

We observe significant variation in skeletal Sr/Ca between different coral genotypes cultured at the same seawater *p*CO_2_ (Fig. 3) demonstrating that other factors also influence Sr incorporation. The origin of this is currently unknown. The mucus layer at the coral surface behaves as a Donnan matrix, concentrating Ca^2+^ from seawater prior to transport across the coral ectoderm tissue^44^ and matrix variations between corals could conceivably subtly influence the Sr/Ca of seawater transported to the calcification fluid. Organic molecules at the calcification site also play a major role in regulating aragonite nucleation and growth^45^. Skeletal organic materials differ between coral species^46^, and may influence skeletal trace element content either because biomolecules impact the incorporation of trace elements in the crystal lattice, or due to direct organic-metal complexing. Understanding the role of these organic templates in fractionating Sr/Ca during calcification, and quantifying differences between coral genotypes even within an individual species, will be a critical step forward in resolving biological influences on skeletal Sr/Ca from the thermal signature.

### Implications for SST reconstructions

Our data show that seawater *p*CO_2_ can be a significant factor affecting the Sr/Ca of massive tropical coral skeletons frequently used for palaeotemperature reconstruction. Decreasing seawater *p*CO_2_ from ambient (416 μatm) to values consistent with the LGM (198 μatm) increases skeletal Sr/Ca by 4% in one of the three coral genotypes. The sensitivity of coral skeletal Sr/Ca to temperature is typically ~0.8–1% °C^−1^ and the effect of the decrease in seawater *p*CO_2_ implies a cooling of 4–5 °C in this single genotype, although all colonies were cultured at the same temperature. The influence of seawater *p*CO_2_ on skeletal Sr/Ca may explain the ‘cold bias’ observed in some fossil corals, which suggest that tropical SSTs at the last or penultimate deglaciations in the Atlantic^47^ and Pacific^48^ Oceans were ~6 °C cooler than at present. These estimates contrast with other marine proxies which indicate that SSTs typically cooled by 2–3 °C at most at this time^49^,^50^. Proposed variations in seawater Sr/Ca over glacial-interglacial periods are insufficient to explain the ‘cold bias’ observed in coral SST reconstruction^20^.

We find that the influence of seawater *p*CO_2_ on skeletal Sr/Ca is inconsistent between corals, even of the same species, and this prevents the calculation of a correction factor to compensate for past variations in seawater *p*CO_2_. Skeletal Sr/Ca is unaffected by *p*CO_2_ over the modern-LGM range relevant to paleotemperature reconstruction in two of our cultured genotypes, yet one genotype shows a substantial, and significant response that is observed across duplicate sub-colonies. We are unable to relate the seawater *p*CO_2_ effect to coral growth rate indicating that coral extension cannot be used to correct skeletal Sr/Ca for an indirect influence of *p*CO_2_ in *Porites* spp., despite successful application of a growth correction in other coral genera^51^. Further culture work is required to resolve the relationship between skeletal Sr/Ca and seawater *p*CO_2_ across a wider range of coral genotypes, and to identify how seawater *p*CO_2_ contributes to ‘cold bias’. At present, the accuracy of absolute tropical past SST estimates from skeletal Sr/Ca of fossil corals is limited by this uncertainty. In contrast, relative changes in annual seawater temperature are usually well preserved in coral skeletal Sr/Ca^52^ and fossil specimens may yet provide good records of Sr/Ca derived seasonal SST variations which can be used to infer the amplitude and frequency of past ENSO^53^ and interdecadal climate events^54^. However, it will also be important to quantify in further studies whether skeletal Sr/Ca temperature sensitivity varies between different coral genotypes, and is affected by seawater *p*CO_2_.

In this study we hypothesise that inconsistencies in SST reconstructions of the LGM from coral Sr/Ca may be explained by a change in seawater *p*CO_2_ between the modern and glacial ocean, equivalent to ~220 μatm. Approximately 50% of this increase in *p*CO_2_ towards present day has occurred in the last 150 years as a result of rising greenhouse gas emissions, overlapping with multi-centennial coral Sr/Ca records. Anthropogenic CO_2_ emissions are isotopically depleted in ^13^C, and a centennial-scale decrease in the δ^13^C of surface ocean DIC attributable to rising atmospheric CO_2_ concentrations^55^ is also observed in the skeletal aragonite of >200 year-old corals collected at the end of the 20^th^ century^56^,^57^. Between 1850 and 2000, the ~85 μatm increase in atmospheric CO_2_ is clearly preserved in the carbon isotopic ratios of these corals, yet there is no discernible influence on skeletal Sr/Ca^5^,^6^. In our culture study, a significant increase in skeletal Sr/Ca was observed at low *p*CO_2_ compared to ambient in one of three genotypes, and we may therefore expect some corals to show a decrease in Sr/Ca with rising CO_2_ over the 20^th^ century. In practice, any CO_2_ forcing on coral Sr/Ca is likely to be obscured by significant interannual variations in SST caused by ENSO against a global trend of increasing SST^5^,^6^,^58^. It is also important to bear in mind that 70% of the increase in atmospheric CO_2_ between 1850 and 2000 occurred in the second half of the twentieth century, so the majority of these multi-centennial coral Sr/Ca records span very small (10–20 μatm) changes in *p*CO_2_. Longer coral Sr/Ca datasets extending into the 21^st^ century are required to investigate the effect of >100 μatm changes in CO_2_ on *in situ* skeletal Sr/Ca.

Our study demonstrates that the variations in published skeletal Sr/Ca-SST calibrations between corals of the same species do not necessarily reflect differences in physical (e.g. temperature, light levels and ocean currents) and chemical (e.g. nutrient levels and composition) conditions^22^,^52^. Rather skeletal Sr/Ca varies significantly between coral genotypes even of the same species cultured under the same conditions. Resolving how genotypic variations affect coral biomineralisation and impact skeletal geochemistry is a key target for future research.

## Methods

### Aquarium System

Culturing experiments were carried out in the marine culturing facility at the Department of Earth & Environmental Sciences, University of St Andrews, UK. Corals were maintained in 21 litre cast acrylic tanks, recirculated from high density polyethylene reservoirs containing ~900 litres of seawater. The reservoirs were bubbled (at 10 L min^−1^) with gas mixes set to reach the target seawater *p*CO_2_ compositions. The modern day treatment was aerated with untreated ambient air. The high and low CO_2_ treatments were aerated with ambient or low-CO_2_ air, respectively, combined with high purity CO_2_ (Foodfresh, BOC, UK). Flow rates of air and CO_2_ were regulated by high-precision mass flow controllers (SmartTrak 50 Series, Sierra USA) controlled by purpose-written MATLAB^®^ programs. Low-CO_2_ air was produced by bubbling ambient air through a caustic solution (0.9 M NaOH and 0.1 M Ca(OH)_2_) and rinsing it by bubbling through deionised water^59^. The [CO_2_] of the low-CO_2_ air (before mixing with CO_2_) was monitored every 2 hours by automated non-dispersive infra-red CO_2_ analysers (WMA04, PP systems, USA) and ranged from 20–100 μatm depending on the age of the caustic solution. The [CO_2_] of the low, ambient and high CO_2_ gas streams (after addition of any CO_2_) was monitored automatically 3–4 times per day and were 180 ± 3, 400 ± 5 and 761 ± 6 μatm (mean ± 1σ) over the experimental period. Corals were maintained under LED lighting (Maxspect R420R 160w–10000k) on a 12 h light: 12 h dark cycle, with wavelength settings of 100% A and 20% B such that light intensity at coral depth was ~300 μmol. Corals were fed weekly with rotifers.

### Monitoring Seawater Composition

Dissolved inorganic carbon (DIC) was measured weekly in each reservoir by LI-7000 CO_2_ differential, non-dispersive, infrared gas analyser (Apollo SciTech; AS-C3). Samples were calibrated against a natural seawater certified reference material (CRM; A. Dickson, Scripps Institution of Oceanography). Internal reproducibility was calculated from the standard deviation of 8 replicate measurements of a single sample (σ/√n), and was always <0.1%. Multiple measurements of the CRM were analysed as unknown samples over the 4 week period to check the calibration, and these were in good agreement with the certified value (unknown = 2019 ± 6 (1σ) μmol kg^−1^, n = 4; CRM = 2014 μmol kg^−1^).

Total alkalinity (TA) was measured twice daily by automated Gran titration (Metrohm, 888 Titrando). Precision of duplicate ~30 ml TA analyses was typically ±2 μeq kg^−1^. Between days, the precision of multiple measurements of synthetic Na_2_CO_3_ standards (1015, 1190, and 1379 μmol kg^−1^) was consistently ±3 μeq kg^−1^ (1σ, n = 14, 10 and 8, respectively). The total alkalinity, [Ca] and [Sr] of the culture seawater was maintained by additions of 0.6 M Na_2_CO_3_ and a mixture of 0.58 M CaCl_2_ + 0.02 M SrCl_2_ by 200 μl volume solenoid diaphragm pumps, evenly spaced over a 24 hour period, controlled by a custom-written MATLAB^®^ dosing control program. We adjusted addition rates to maintain total alkalinity within narrow limits (±≤14 μmol kg^−1^). Total alkalinity variations of this magnitude have little effect on seawater carbonate chemistry (~0.002 pH units, ~0.6% [DIC]) at constant seawater *p*CO_2_.

Seawater samples were collected weekly during the experimental period for Sr and Ca analysis by quadrupole ICP-MS (Thermo Scientific X Series) at the National Oceanography Centre, Southampton. Samples were diluted 1000-fold in 5% HNO_3_ (with 5 ppb In as an internal standard) and calibrated against matrix-matched synthetic standards prepared from 1000 μg ml^−1^ single-element stock solutions (Inorganic Ventures) in 5% HNO_3_. Based on replicate analyses (n = 4) of IAPSO standard seawater, external reproducibility (σ/√n) was 0.6% for Sr/Ca. Measured values of Sr/Ca in IAPSO (8.78 ± 0.05 (1σ) mmol mol^−1^) were in excellent agreement with certified values (8.77 mmol mol^−1^).

Nutrients were measured in filtered (Whatman GFF; ~0.7 μm) seawater samples from each reservoir by flow cell spectrophotometry (Lachat 8000; Scottish Association of Marine Science). Concentrations of NH_4_^+^ (<0.19 ± 0.01 μmol L^−1^; mean ± 1σ), PO_4_^3−^ (<0.06 ± 0.00 μmol L^−1^), SiO_4_^4−^ (<1.3 ± 0.03 μmol L^−1^) and NO_3_ + NO_2_ (<0.95 ± 0.02 μmol L^−1^) were within the range reported for pristine reef waters^60^.

### Coral sample processing and SIMS

Skeletal samples were prepared from the maximum growth axis and fixed in epoxy resin (Epo thin, Struers UK) in 2.5 cm diameter circular moulds, under vacuum. Polished, gold coated sections were analysed using a Cameca imf-4f ion microprobe in the School of Geosciences at the University of Edinburgh (^16^O^−^ ion beam accelerated at 10.8 kV; primary beam current = 8 nA; energy offset = 75 eV; field aperture 1; contrast aperture 2). Trace element data were collected over 8 days, and multiple analyses of a fossil coral aragonite standard, NaHaxby2 (Sr/Ca = 2.87 mmol mol^−1^), were made each day. Calculation of relative ion yields (RIYs) and standardisation of data was performed as in Allison *et al.*^16^. Internal reproducibility was calculated from ten cycles of a single SIMS analysis (2σ/√10) and was ~0.4% for Sr/Ca. External reproducibility (the precision of ~15 daily analyses on the standard) was 0.6%, or 0.018 mmol mol^−1^, (2σ) for Sr/Ca. Where significant variations in the RIY for NaHaxby2 were observed between days, instrument drift was distinguished from heterogeneity in the composition of the standard through additional analyses of the same area on a subsequent day. Samples were calibrated against mean standard RIYs, either from daily or weekly analyses depending on this assessment of instrument performance. See Allison *et al.*^32^ for representative images of skeletal ultrastructure and SIMS analyses pits^32^.

Daily calcification rates (μmol CaCO_3 _cm^−2^ d^−1^) were estimated for each sub colony from the measured change in seawater TA over an isolation period in the light (5 hours), and the dark (7 hours), on three separate occasions over the experimental period. Linear extension (μm) was measured as the distance between the alizarin stain lines in each analysed trabecula.

## Additional Information

**How to cite this article**: Cole, C. *et al.* Understanding cold bias: Variable response of skeletal Sr/Ca to seawater *p*CO_2_ in acclimated massive Porites corals. *Sci. Rep.* **6**, 26888; doi: 10.1038/srep26888 (2016).

## Supplementary Material

Supplementary Information

## Figures and Tables

**Figure 1:**
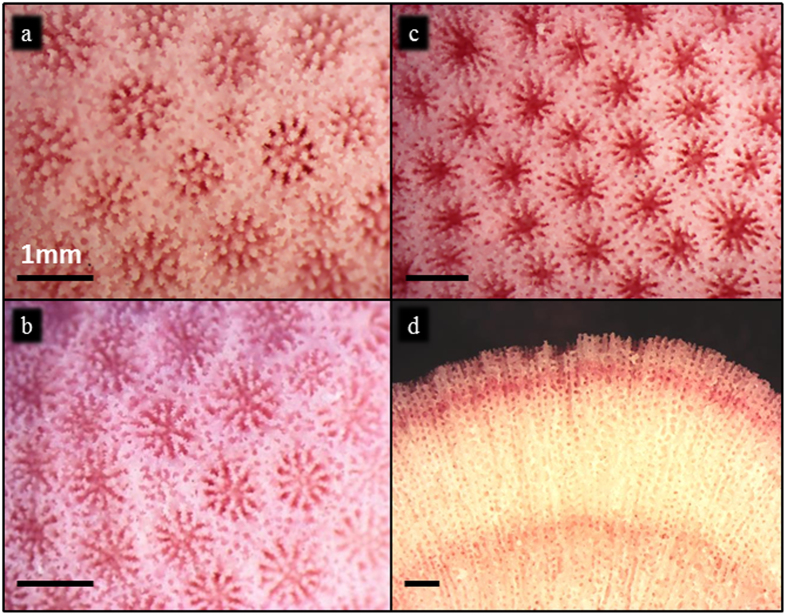
Multiple genotypes of massive *Porites* spp. stained with alizarin. Representative colonies of (**a**) *Porites lutea* genotype 1; (**b**) *Porites lutea* genotype 2; (**c**) *Porites murrayensis*, cultured at ambient *p*CO_2_ within our aquarium system. (**d**) Alizarin stain lines mark skeletal extension during the 5-week experimental period. Scale bars are 1mm.

**Table 1:** Summary of the physical and chemical composition of seawater in each culture treatment, measured over the 5-week experimental period.

Treatment	Sr/Ca (mmol/mol)	Temperature (°C)	Salinity	TA (μeq/kg)	DIC (μmol/kg)	pH_T_	*p*CO_2_ (μatm)	HCO_3_^−^(μmol/kg)	CO_3_^2−^(μmol/kg)	Ω_Ar_
Last Glacial Maximum	12.27 ± 0.09	25.3 ± 0.1	35.1 ± 0.04	2289 ± 14	1831 ± 11	8.28	198	1511	315	5.01
Modern Day	12.07 ± 0.03	25.2 ± 0.2	35.2 ± 0.08	2290 ± 8	1999 ± 8	8.03	416	1780	207	3.29
Future (~Year 2100)	11.85 ± 0.03	25.1 ± 0.2	35.2 ± 0.05	2293 ± 5	2113 ± 11	7.81	750	1954	138	2.19

Values are the mean ± 1σ. Dissolved inorganic carbon (DIC) and Total Alkalinity (TA) were used to calculate seawater carbonate system components (pH_T_, *p*CO_2_, HCO_3_^−^, CO_3_^2−^ and Ω_Ar_) using CO2SYS (Pierrot *et al.*^30^, whereby pH is Total scale; K_1_ and K_2_ are from Mehrbach *et al.*^61^ refit by Dickson and Millero; K_SO4_ as determined by Dickson.

## References

[b1] Beck J. W. *et al.* Sea-surface temperature from coral skeletal strontium/calcium ratios. Science 257, 644–647 (1992). 17740731 10.1126/science.257.5070.644

[b2] Quinn T. M. & Sampson D. E. A multiproxy approach to reconstructing sea surface conditions using skeleton geochemistry. Paleoceanography 17, 1062, 10.1029/2000PA000528 (2002).

[b3] Linsley B. K. *et al.* Geochemical evidence from corals for changes in the amplitude and spatial pattern of South Pacific interdecadal climate variability over the last 300 years. Clim. Dynam. 22, 1–11 (2004).

[b4] Stephans C. L., Quinn T. M., Taylor F. W. & Corrège T. Assessing the reproducibility of coral-based climate records. Geophys. Res. Lett. 31, L18210, 10.1029/2004GL020343 (2004).

[b5] DeLong K. L., Quinn T. M., Taylor F. W., Lin K. & Shen C.-C. Sea surface temperature variability in the southwest tropical Pacific since AD 1649. Nat. Clim. Change 2, 799–804 (2012).

[b6] Hendy E. J. *et al.* Abrupt decrease in tropical Pacific sea surface salinity at the end of the Little Ice Age. Science 295, 1511–1514 (2002). 11859191 10.1126/science.1067693

[b7] Linsley B. K., Wellington G. M. & Schrag D. P. Decadal sea surface temperature variability in the Subtropical South Pacific from 1726 to 1997 A. D. Science 290, 1145–1148 (2000). 11073450 10.1126/science.290.5494.1145

[b8] Beck J. W., Récy J., Taylor F. W., Lawrence Edwards R. & Cabioch G. Abrupt changes in early Holocene tropical sea surface temperature derived from coral records. Nature 385, 705–707 (1997).

[b9] Corrège T. *et al.* Interdecadal variaion in the extent of South Pacific tropical waters during the Younger Dryas event. Nature 428, 927–929 (2004). 15118722 10.1038/nature02506

[b10] DeLong K. L., Quinn T. M., Shen C.-C. & Lin K. A snapshot of climate variability at Tahiti at 9.5 ka using a fossil coral from IODP Expedition 310. Geochem. Geophys. Geosyst. 11, 10.1029/2009GC002758 (2010).

[b11] Asami R. *et al.* Evidence for tropical South Pacific climate change during the Younger Dryas and the Bølling–Allerød from geochemical records of fossil Tahiti corals. Earth Planet. Sci. Lett. 288, 96–107 (2009).

[b12] de Villiers S., Nelson B. K. & Chivas A. R. Biological controls on coral Sr/Ca and δ^18^O reconstructions of sea surface temperatures. Science 269, 1247–1249 (1995). 17732111 10.1126/science.269.5228.1247

[b13] Kuffner I. B., Jokiel P. L. & Rodgers K. S. An apparent “vital effect” of calcification rate on the Sr/Ca temperature proxy in the reef coral *Montipora capitata*. Geochem. Geophys. Geosyst. 13, 10.1029/2012GC004128 (2012).

[b14] Allison N. & Finch A. A. High-resolution Sr/Ca records in modern *Porites lobata* corals: Effects of skeletal extension rate and architecture. Geochem. Geophys. Geosyst. 5, 10.1029/2004GC000696 (2004).

[b15] Hayashi E. *et al.* Growth-rate influences on coral climate proxies tested by a multiple colony culture experiment. Earth Planet. Sci. Lett. 362, 198–206 (2013).

[b16] Allison N., Finch A. A., Webster J. M. & Clague D. A. Palaeoenvironmental records from fossil corals: The effects of submarine diagenesis on temperature and climate estimates. Geochim. Cosmochim. Acta 71, 4693–4703 (2007).

[b17] Allison N. *et al.* Reconstruction of deglacial sea surface temperatures in the tropical Pacific from selective analysis of a fossil coral. Geophys. Res. Lett. 32, 10.1029/2005GL023183 (2005).

[b18] Allison N. Geochemical anomalies in coral skeletons and their possible implications for palaeoenvironmental analyses. Mar. Chem. 55, 367–379 (1996).

[b19] Meibom A. *et al.* Monthly strontium/calcium oscillations in symbiotic coral aragonite: Biological effects limiting the precision of the paleotemperature proxy. Geophys. Res. Lett. 30, 10.1029/2002GL016864 (2003).

[b20] Stoll H. M. & Schrag D. P. Effects of Quaternary sea level cycles on strontium in seawater. Geochim.Cosmochim. Acta 62, 1107–1118 (1998).

[b21] Sinclair D. J. RBME coral temperature reconstruction: An evaluation, modification, and recommendations. Geochim. Cosmochim. Acta 154, 66–80 (2015).

[b22] Reynaud S. *et al.* Light and temperature effects on Sr/Ca and Mg/Ca ratios in the scleractinian coral *Acropora* sp. Geochim. Cosmochim. Acta 71, 354–362 (2007).

[b23] Petit J. R. *et al.* Climate and atmospheric history of the past 420,000 years from the Vostok ice core, Antarctica. Nature 399, 429–436 (1999).

[b24] Hönisch B. & Hemming N. G. Surface ocean pH response to variations in *p*CO_2_ through two full glacial cycles. Earth Planet. Sci. Lett. 236, 305–314 (2005).

[b25] Dissard D., Nehrke G., Reichart G. J. & Bijma J. Impact of seawater *p*CO_2_ on calcification and Mg/Ca and Sr/Ca ratios in benthic foraminifera calcite: results from culturing experiments with *Ammonia tepida*. Biogeosciences 7, 81–93 (2010).

[b26] Tanaka K. *et al.* Response of *Acropora digitifera* to ocean acidification: constraints from δ^11^B, Sr, Mg, and Ba compositions of aragonitic skeletons cultured under variable seawater pH. Coral Reefs, 10.1007/s00338-015-1319-6 (2015).

[b27] Cohen A. L., McCorkle D. C., de Putron S., Gaetani G. A. & Rose K. A. Morphological and compositional changes in the skeletons of new coral recruits reared in acidified seawater: Insights into the biomineralization response to ocean acidification. Geochem. Geophys. Geosyst. 10, 10.1029/2009GC002411 (2009).

[b28] Gagnon A. C., Adkins J. F., Erez J., Eiler J. M. & Guan Y. Sr/Ca sensitivity to aragonite saturation state in cultured subsamples from a single colony of coral: Mechanism of biomineralization during ocean acidification. Geochim. Cosmochim. Acta 105, 240–254 (2013).

[b29] Gattuso J.-P. & Lavigne H. Technical Note: Approaches and software tools to investigate the impact of ocean acidification. Biogeosciences 6, 2121–2133 (2009).

[b30] Pierrot D., Lewis E. & Wallace D. W. R. MS Excel program developed for CO_2_ system calculations, ORNL/CDIAC-105a. Carbon Dioxide Information Analysis Center, Oak Ridge National Laboratory, US Department of Energy, Tennessee, USA. URL http://cdiac.ornl.gov/oceans/co2rprt.html (2006).

[b31] Barnes D. J. & Lough J. M. On the nature and causes of density banding in massive coral skeletons. J. Exp. Mar. Biol. Ecol. 167, 91–108 (1993).

[b32] Allison N. & Finch A. A. & EIMF. δ^11^B, Sr, Mg and B in a modern *Porites* coral: the relationship between calcification site pH and skeletal chemistry. Geochim. Cosmochim. Acta 74, 1790–1800 (2010).

[b33] Marshall A. T. Calcification in hermatypic and ahermatypic corals. Science 271, 637–639 (1996).

[b34] Tambutté S., Allemand D. & Jaubert J. Permeability of the oral epithelial layers in cnidaians. Mar. Biol. 126, 43–53 (1996).

[b35] Ip Y. K. & Lim A. L. L. Are calcium and strontrium transported by the same mechanism in the hermatypic coral *Galaxea fascicularis?* J. Exp. Biol. 159, 507–513 (1991).

[b36] Venn A. A. *et al.* Impact of seawater acidification on pH at the tissue–skeleton interface and calcification in reef corals. Proc. Natl. Acad. Sci. USA 110, 1634–1639 (2013). 23277567 10.1073/pnas.1216153110 PMC3562847

[b37] Al-Horani F. A., Al-Moghrabi S. M. & de Beer D. The mechanism of calcification and its relation to photosynthesis and respiration in the scleractinian coral *Galaxea fascicularis*. Mar. Biol. 142, 419–426 (2003).

[b38] Allison N., Cohen I., Finch A. A. & Erez J. & EMIF. Controls on Sr/Ca and Mg/Ca in scleractinian corals: The effects of Ca-ATPase and transcellular Ca channels on skeletal chemistry. Geochim. Cosmochim. Acta 75, 6350–6360 (2011).

[b39] Gaetani G. A. & Cohen A. L. Element partitioning during precipitation of aragonite from seawater: A framework for understanding paleoproxies. Geochim. Cosmochim. Acta 70, 4617–4634 (2006).

[b40] Elderfield H., Bertram C. J. & Erez J. A biomineralization model for the incorporation of trace elements into foraminiferal calcium carbonate. Earth Planet. Sci. Lett. 142, 409–423 (1996).

[b41] Holcomb M. *et al.* Coral calcifying fluid pH dictates response to ocean acidification. Sci. Rep. 4, 10.1038/srep05207 (2014). PMC4047535 24903088

[b42] Allison N. *et al.* Corals concentrate dissolved inorganic carbon to facilitate calcification. Nat. Commun. 5, 10.1038/ncomms6741 (2014). 25531981

[b43] Williamson P. & Turley C. Ocean acidification in a geoengineering context. Phil. Trans. R. Soc. A 370, 4317–4342 (2012). 22869801 10.1098/rsta.2012.0167 PMC3405667

[b44] Marshall A. T. & Clode P. L. Light-regulated Ca^2+^ uptake and O_2_ secretion at the surface of a scleractinian coral *Galaxea fascicularis*. Comp. Biochem. Physiol. A 136, 417–426 (2003). 10.1016/s1095-6433(03)00201-0 14511760

[b45] Tambutté S. *et al.* Coral biomineralization: From the gene to the environment. J. Exp. Mar. Biol. Ecol. 408, 58–78 (2011).

[b46] Falini G. *et al.* Control of aragonite deposition in colonial corals by intra-skeletal macromolecules. J. Struct. Biol. 183, 226–238 (2013). 23669627 10.1016/j.jsb.2013.05.001

[b47] Guilderson T. P., Fairbanks R. & Rubenstone J. L. Tropical temperature variations since 20,000 years ago: Modulating interhemispheric climate change. Science 263, 663–665 (1994). 17747661 10.1126/science.263.5147.663

[b48] McCulloch M. T. *et al.* Coral record of equatorial sea-surface temperatures during the penultimate deglaciation at Huon Peninsula. Science 283, 202–204 (1999). 9880248 10.1126/science.283.5399.202

[b49] Lea D. W., Pak D. K. & Spero H. J. Climate impact of late quaternary equatorial Pacific sea surface temperature variations. Science 289, 1719–1724 (2000). 10976060 10.1126/science.289.5485.1719

[b50] Visser K., Thunell R. & Stott L. Magnitude and timing of temperature change in the Indo-Pacific warm pool during deglaciation. Nature 421, 152–155 (2003). 12520298 10.1038/nature01297

[b51] Goodkin N. F., Hughen K. A. & Cohen A. L. A multicoral calibration method to approximate a universal equation relating Sr/Ca and growth rate to sea surface temperature. Paleoceanography 22, 10.1029/2006PA001312 (2007).

[b52] McCulloch M. T. & Esat T. The coral record of last interglacial sea levels and sea surface temperatures. Chem. Geol. 169, 107–129 (2000).

[b53] Gagan M. K., Hendy E. J., Haberle S. G. & Hantoro W. S. Post-glacial evolution of the Indo-Pacific Warm Pool and El Niño-Southern oscillation. Quat. Int. 118–119, 127–143 (2004).

[b54] Calvo E. *et al.* Interdecadal climate variability in the Coral Sea since 1708 A. D. Palaeogeogr. Palaeoclimatol. Palaeoecol. 248, 190–201 (2007).

[b55] Druffel E. R. M. & Benavides L. M. Input of excess CO_2_ to the surface ocean based on ^13^C/^12^C ratios in a banded Jamaican sclerosponge. Nature 321, 58–61 (1986).

[b56] Swart P. K. *et al.* The ^13^C Suess effect in scleractinian corals mirror changes in the anthropogenic CO_2_ inventory of the surface oceans. Geophys. Res. Lett. 37, L05604, 10.1029/2009GL041397 (2010).

[b57] Dassié E. P., Lemley G. M. & Linsley B. K. The Suess effect in Fiji coral δ^13^C and its potential as a tracer of anthropogenic CO_2_ uptake. Palaeogeogr. Palaeoclimatol. Palaeoecol. 370, 30–40 (2013).

[b58] DeLong K. L., Quinn T. M. & Taylor F. W. Reconstructing twentieth-century sea surface temperature variability in the southwest Pacific: A replication study using multiple coral Sr/Ca records from New Caledonia. Paleoceanography 22, PA4212, 10.1029/2007PA001444 (2007).

[b59] Hintz C. J. Inventor; University of South Carolina, assignee. High efficiency, non-toxic scrubbing system and method for removing carbon dioxide from a gas. United States patent US 8,167,979 B2. 2012 May 1.

[b60] Furnas M., Alongi D., McKinnon D., Trott L. & Skuza M. Regional-scale nitrogen and phosphorus budgets for the northern (14°S) and central (17°S) Great Barrier Reef shelf ecosystem. Cont. Shelf Res. 31, 1967–1990 (2011).

[b61] Mehrbach, C., Culberson C. H., Hawley, J. E. & Pytkowicz, R. M. Measurement of the apparent dissociation constants of carbonic acid in seawater at atmospheric pressure. Limnol. Oceanogr. 18, 897–907 (1973).

