# Supplementary material for: Understanding cold bias: Variable response of skeletal Sr/Ca to seawater pCO2 in acclimated massive Porites corals
Source: Sci Rep. 2016 May 31;6:26888. doi: 10.1038/srep26888 (PMC4886260; doi:10.1038/srep26888)
Supplement: Supplementary Information [file srep26888-s1.pdf]

Understanding cold bias: Variable response of skeletal Sr/Ca to seawater pCO_2 in acclimated massive Porites corals

Catherine Cole *[1], Adrian Finch [1], Christopher Hintz [2], Kenneth Hintz [3], Nicola Allison [1]

Table S1. Measured growth and Sr/Ca in individual skeletal units within colonies cultured across a range of seawater pCO_2 conditions representing the Last Glacial Maximum (LGM; pH 8.3), modern day (ambient; pH 8.0) and projected levels for the year 2100 (pH 7.8). Linear extension was measured between the two stain lines marking skeletal growth during the 5 week experimental period. Following SIMS, molar Sr/Ca ratios in the skeleton, and partition coefficients between aragonite and seawater ($K_D^{Sr/Ca}$), are reported with 95% confidence intervals (95% CI) and the number of analyses per unit (n).

Treatment	Species/ Genotype	Colony ID	Skeletal Unit	Linear Extension (µm)	Sr/Ca (µmol mol^{-1})	*95% CI*	$K_D^{Sr/Ca}$	*95% CI*	n
**LGM**	*P. lutea* Gen 1	M3	A	1260	11.90	*0.08*	0.971	*0.010*	19
			B	1380	11.98	*0.09*	0.977	*0.011*	22
	P. lutea Gen 2	H1	A	770	12.11	*0.27*	0.988	*0.024*	10
			B	780	12.17	*0.19*	0.993	*0.018*	14
			C	580	12.02	*0.41*	0.981	*0.034*	4
	P. lutea Gen 2	C1	A	770	12.26	*0.21*	1.001	*0.019*	9
			B	540	12.10	*0.16*	0.988	*0.015*	7
			C	450	11.90	*0.19*	0.971	*0.018*	5
			D	460	12.38	*0.19*	1.010	*0.018*	5
	P. murrayensis	X1	A	500	11.80	*0.06*	0.963	*0.009*	6
			B	360	12.05	*0.17*	0.983	*0.016*	9
			C	480	11.70	*0.12*	0.955	*0.012*	5
**Ambient**	*P. lutea* Gen 1	M1	A	1120	11.78	*0.11*	0.977	*0.009*	17
			B	840	11.68	*0.14*	0.969	*0.012*	9
	P. lutea Gen 2	H2	A	450	11.52	*0.12*	0.955	*0.010*	7
			B	460	11.48	*0.34*	0.952	*0.028*	6
			C	420	11.36	*0.19*	0.940	*0.016*	7
			D	380	11.64	*0.32*	0.966	*0.026*	3
	P. murrayensis	X2	A	750	11.86	*0.15*	0.984	*0.013*	13
			B	760	11.70	*0.23*	0.971	*0.019*	10
	P. murrayensis	U	A	930	11.79	*0.09*	0.977	*0.008*	14
			B	730	11.72	*0.13*	0.972	*0.011*	6
**Year 2100**	*P. lutea* Gen 1	M2	A	480	12.02	*0.19*	1.016	*0.016*	8
			B	580	11.95	*0.19*	1.009	*0.016*	9
			C	390	11.87	*0.19*	1.002	*0.016*	3
			D	430	11.90	*0.27*	1.005	*0.023*	4
	P. lutea Gen 2	B2	A	230	11.32	*0.25*	0.956	*0.021*	5
			B	230	11.46	*0.16*	0.968	*0.014*	5
			C	180	11.65	*0.22*	0.984	*0.019*	7
			D	210	11.51	*0.42*	0.972	*0.036*	4
	P. murrayensis	A	A	620	11.58	*0.19*	0.978	*0.016*	6
			B	370	11.62	*0.18*	0.981	*0.015*	6
	P. murrayensis	V	A	480	11.76	*0.17*	0.993	*0.015*	8
			B	440	11.50	*0.14*	0.971	*0.012*	7

Table S2. Mean linear extension and daily calcification rate in three genotypes of *Porites* spp. cultured at 198, 416, and 750 µatm pCO_2 after >5 months of acclimation.

Seawater pCO_2 (µatm)	*P. lutea* Genotype 1	*P. lutea* Genotype 2	*P. murrayensis*
Linear Extension (µm; mean ± 1σ)			
198	1320 ± 90^{a}	710 ± 110^{a} and 550 ± 140^{ab}	450 ± 80^{a}
416	980 ± 200^{b}	430 ± 40^{b}	760 ± 10^{ab} and 830 ± 140^{b}
750	470 ± 80^{c}	210 ± 20^{c}	460 ± 30^{ab} and 500 ± 180^{ab}
Daily calcification rate (µmol $CaCO_3$ cm^{-2} d^{-1}; mean ± 1σ)			
198	21 ± 1^{a}	21 ± 3^{a} and 14 ± 1^{b}	23 ± 1^{a}
416	19 ± 1^{a}	10 ± 2^{b}	15 ± 1^{bc} and 17 ± 1^{ab}
750	18 ± 3^{a}	4 ± 1^{c}	11 ± 2^{bc} and 9 ± 4^{cd}

Linear extension measurements represent the means of 2+ skeletal units over the 5 week experimental period; calcification rates are the mean of 3 weekly whole-colony measurements. Duplicate colonies shown for *P. lutea* Genotype 2 (198 µatm) and *P. murrayensis* (416 and 750 µatm). Within each species/genotype, different letters indicate significant differences between treatments ($p < 0.05$; ANOVA and Tukey post-hoc).

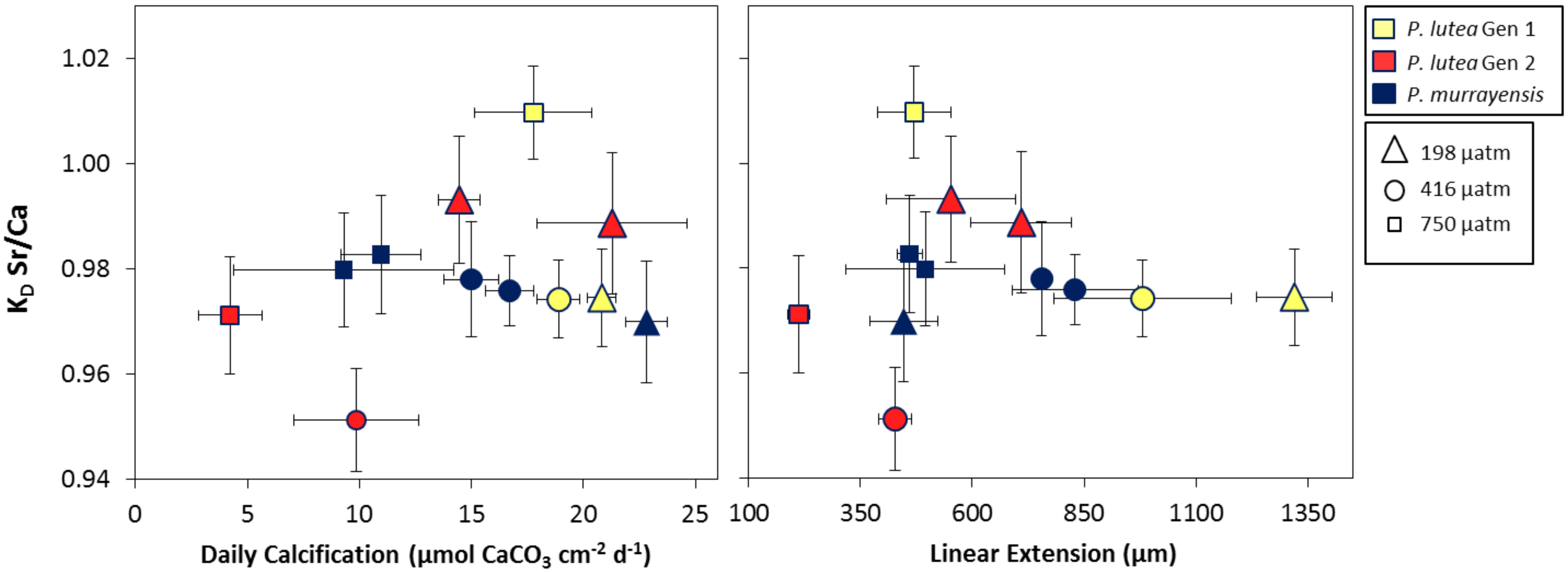

Figure S1. Mean $K_D^{Sr/Ca}$ of *Porites* spp. colonies plotted against mean daily calcification rate and linear extension. Corals cultured at 198, 416 and 750 µatm pCO_2 are indicated by symbols defined in the key. Error bars are combined 95% confidence limits of seawater and skeletal Sr/Ca measurements ($K_D^{Sr/Ca}$); 1σ of 3 weekly calcification rate measurements; and 1σ of linear extension measurements between skeletal units.
